# Structural and Functional Characterization of the Most Frequent Pathogenic PRKN Substitution p.R275W

**DOI:** 10.3390/cells13181540

**Published:** 2024-09-13

**Authors:** Bernardo A. Bustillos, Liam T. Cocker, Mathew A. Coban, Caleb A. Weber, Jenny M. Bredenberg, Paige K. Boneski, Joanna Siuda, Jaroslaw Slawek, Andreas Puschmann, Derek P. Narendra, Neill R. Graff-Radford, Zbigniew K. Wszolek, Dennis W. Dickson, Owen A. Ross, Thomas R. Caulfield, Wolfdieter Springer, Fabienne C. Fiesel

**Affiliations:** 1 Mayo Clinic, Department of Neuroscience, Jacksonville, FL 32224, USA; bernardo.bustillos@mayo.edu (B.A.B.); liamthomascocker2000@gmail.com (L.T.C.); coban.mathew@mayo.edu (M.A.C.); weber.caleb@mayo.edu (C.A.W.); bredenbergj@gmail.com (J.M.B.); dickson.dennis@mayo.edu (D.W.D.); ross.owen@mayo.edu (O.A.R.); caulfield.thomas@mayo.edu (T.R.C.); 2 Department of Neurology, Faculty of Medical Sciences in Katowice, Medical University of Silesia, 40-055 Katowice, Poland; 3 Department of Neurology, St. Adalbert Hospital, 80-462 Gdansk, Poland; jaroslaw.slawek@gumed.edu.pl; 4 Division of Neurological and Psychiatric Nursing, Faculty of Health Sciences, Medical University of Gdansk, 80-210 Gdansk, Poland; 5 Department of Clinical Sciences, Neurology, Lund University, 22100 Lund, Sweden; andreas.puschmann@med.lu.se; 6 Department of Neurology, Skane University Hospital, 22185 Lund, Sweden; 7 Inherited Movement Disorders Unit, Neurogenetics Branch, National Institute of Neurological Disorders and Stroke (NINDS), NIH, Bethesda, MD 20892, USA; derek.narendra@nih.gov; 8 Mayo Clinic, Department of Neurology, Jacksonville, FL 32224, USA; graffradford.neill@mayo.edu; 9 Mayo Clinic, Graduate School of Biomedical, Sciences Neuroscience PhD Program, Jacksonville, FL 32224, USA; wszolek.zbigniew@mayo.edu; 10 Mayo Clinic, Department of Neurosurgery, Jacksonville, FL 32224, USA; 11 Mayo Clinic, Department of Cancer Biology, Jacksonville, FL 32224, USA; 12 Mayo Clinic, Department of Biochemistry & Molecular Biology, Jacksonville, FL 32224, USA; 13 Mayo Clinic, Department of Computational Biology, Jacksonville, FL 32224, USA

**Keywords:** mitophagy, parkin, Parkinson disease, PINK1, PRKN, ubiquitin

## Abstract

Mutations in the *PINK1* and *PRKN* genes are the most frequent genetic cause of early-onset Parkinson disease. The pathogenic p.R275W substitution in PRKN is the most frequent substitution observed in patients, and thus far has been characterized mostly through overexpression models that suggest a possible gain of toxic misfunction. However, its effects under endogenous conditions are largely unknown. We used patient fibroblasts, isogenic neurons, and post-mortem human brain samples from carriers with and without PRKN p.R275W to assess functional impact. Immunoblot analysis and immunofluorescence were used to study mitophagy activation, and mitophagy execution was analyzed by flow cytometry of the reporter mitoKeima. The functional analysis was accompanied by structural investigation of PRKN p.R275W. We observed lower PRKN protein in fibroblasts with compound heterozygous p.R275W mutations. Isogenic neurons showed an allele-dose dependent decrease in PRKN protein. Lower PRKN protein levels were accompanied by diminished phosphorylated ubiquitin and decreased MFN2 modification. Mitochondrial degradation was also allele-dose dependently impaired. Consistently, PRKN protein levels were drastically reduced in human brain samples from p.R275W carriers. Finally, structural simulations showed significant changes in the closed form of PRKN p.R275W. Our data suggest that under endogenous conditions the p.R275W mutation results in a loss-of-function by destabilizing PRKN.

## 1. Introduction

The pathology of Parkinson disease (PD), the second most common neurodegenerative disease, is characterized by the formation of α-synuclein aggregates in the form of Lewy bodies (LB) and the selective loss of dopaminergic (DA) neurons in the substantia nigra [1]. Loss-of-function mutations in *PINK1* and *PRKN* are the most common genetic cause of the early-onset form of PD, defined as onset before age 50 years. The encoded proteins cooperate to selectively identify and eliminate damaged mitochondria by autophagy (mitophagy) [2,3,4]. Upon mitochondrial depolarization, PINK1, a mitochondrial kinase, swiftly accumulates on the outer mitochondrial membrane and phosphorylates ubiquitin (Ub) [5,6,7] and the E3 Ub ligase PRKN at a conserved serine-65 [7,8,9]. These events are needed for the recruitment of PRKN from the cytosol to mitochondria and for its activation. Mitochondrial, activated PRKN attaches more Ub molecules that serve as additional substrates for PINK1 [10]. This positive feedback loop ensures the timely decoration of damaged mitochondria with phosphorylated (pS65-) Ub chains, a label that is recognized by specific autophagy receptors and facilitates uptake of the damaged mitochondria by autophagosomes for their degradation in lysosomes [11].

Among the pathogenic point mutations that have been identified in PRKN, c.823C > T (rs34424986; p.R275W) is the most common missense variant [12,13]. Clinically, patients carrying the p.R275W substitution present with typical, early-onset PD with a slow progression, good response to low doses of levodopa, and the absence of dementia [14]. While LBs were remarkably absent in most autopsy studies from patients with *PRKN* mutations, some individuals with PRKN p.R275W presented with LB pathology [15,16,17]. In addition, and in contrast to other loss-of-function mutations, various functional studies overexpressing PRKN p.R275W have found that it retains its ability to ubiquitylate some substrates [18,19], and that it has a propensity to form aggresomes [19,20,21,22]. This led to the hypothesis that residual Ub ligase function or a gain of misfunction might be required for LB formation [16,23]. However, most of the functional studies have used recombinant or overexpressed PRKN p.R275W.

PRKN is an RBR-type E3 Ub ligase that is typically kept autoinhibited through several intramolecular interactions [24]. Apart from the N-terminal Ub-like (UBL) domain, which maintains PRKN in a closed state, its functionality is inhibited by the REP (repressor of PRKN) segment, obstructing the E2 binding site within its typical RING1 configuration. Furthermore, RING0 physically separates RING1 and RING2. This physical separation causes RING2, the location of the active site, to attach to RING0 instead of RING1, which further inhibits the transfer of Ub. Analysis of the closed structure reveals that PRKN critically needs activation and significant structural rearrangements to acquire enzymatic E3 Ub ligase activity [24]. PRKN activation is initiated by binding to pS65-Ub with the RING1 domain and, after molecular rearrangement, phosphorylation by PINK1 at S65. In this activated form of PRKN, mutation of p.R275 to a tryptophan (W) is expected to lead to clashes with both the nearby helix in the RING1 and residues of the UBL domain, thereby ultimately resulting in destabilization of PRKN [25].

Here, we investigated the function of PRKN p.R275W under endogenous conditions in patients’ cells, in isogenic dopaminergic neurons, the latter generated by CRISPR–Cas9 and in human post-mortem brain samples. In contrast to wild-type (WT) PRKN, we found that the protein levels of endogenous PRKN p.R275W were strongly decreased. Consistently, cells with the p.R275W mutation presented with lower mitophagy initiation and execution upon mitochondrial stress. On the structural level, we found evidence of destabilization of the closed PRKN conformation. Together, our data suggest that p.R275W renders the PRKN protein unstable, and results in a complete loss of function due to loss of the PRKN protein.

## 2. Material and Methods

### 2.1. Structural Analysis

For this study, we utilized our previously published full-length homology model of PRKN WT [26]. This model consists of residues 1-465 of human Parkin/PRKN, with eight zincs bound, and is based on the available experimental structures of Parkin (UniProt accession #O60260). Homology model construction was utilized to complete stretches of missing residues, such as the linker region. The complete model was generated using YASARA [27,28], which was previously selected over models generated via other systems such as AlphaFold [26]. Using the PRKN WT model as a template, p.R275W PRKN was generated by the mutagenesis wizard in PyMOL [The PyMOL Molecular Graphics System, Version 2.5.2, Schrödinger, LLC, New York, NY, USA]. We then conducted identical all—atom unbiased molecular dynamics simulations (MDS) on PRKN WT and p.R275W that were run side—by—side using the following conditions: Rectangular simulation boxes were constructed with edges extending 18 Å from the nearest protein atom, which was filled with 0.997 g/L TIP3P waters and 150 mM (0.9%) Na+/Cl− ions at a pH of 7.4. System temperature (310 K) and pressure (1 bar) were maintained around their average values via coupling to computational thermostat/barostat [29,30]. Long range electrostatics were calculated using the particle mesh Ewald method with periodic boundary of 7.86 Å [31]. Under these conditions, PRKN variants were subjected to energy minimization using the steepest descent Polak–Ribière conjugate gradient method for 100 ps [32]. The Amber14ff was utilized with a 2.5 fs timestep with holonomic X-H bond restraints using the SHAKE algorithm, with post-equilibrated analytical simulations extending for 1 microsecond [33]. MDTraj was used to perform root mean square deviation (RMSD) and root mean square fluctuation (RMSF) calculations. The nearest-neighbor function of MDTraj was used to determine residues located within 5 Å of the RING1 binding site [34]. Visualization and structure comparisons were performed with PyMOL and BioLuminate (https://www.schrodinger.com/platform/products/bioluminate/, accessed on 14 July 2024).

### 2.2. Cell Culture, Treatment and Harvest

Human fibroblasts originating from *PRKN* (c.823C > T) mutation carriers encoding for PRKN p.R275W and age/sex-matched control (WT) fibroblasts were grown in Dulbecco’s Modified Eagle Medium (DMEM [Thermo Fisher Scientific, Waltham, MA, USA, 11965118]) containing 10% heat-inactivated Fetal Bovine Serum (FBS, [Neuromics, Edina, MN, USA, FBS001800112]), 1% non-essential amino acids (Invitrogen, Waltham, MA, USA, 11140-050), and 0.5% PenStrep (Thermo, 15140122). The collection and use of skin fibroblasts was approved by the Institutional Review Board at Mayo Clinic. Some fibroblasts were obtained from the NINDS Human Cell and Data Repository (NHCDR, ND29510, ND29369). A compound heterozygous PRKN p.R275W fibroblast line was obtained from Dr. Derek Narendra, NIH.

ReNcell VM (Millipore, Burlington, MA, USA, SCC008), an immortalized human neural progenitor cell line, was transduced with lentivirus encoding mitochondrially targeted mKeima (mitoKeima). Upon selection with blasticidin, cells were sorted three times by flow cytometry in order to generate a population with homogenous mitoKeima expression levels. ReN VM mitoKeima cells were cultured in DMEM/F12 media (Thermo Fisher Scientific, 11320033), supplemented with 2% B-27 with antioxidants (Thermo Fisher Scientific, 17504044) containing 10 U/mL Heparin (Sigma, St. Louis, MA, USA, H3149) and 0.1% Gentamicin (Thermo Fisher Scientific, 15-750-060). The media was further supplemented with 20 µg/mL fibroblast growth factor (FGF, Peprotech, 100-25) and 20 µg/mL epidermal growth factor (EGF, Peprotech, Cranbury, NJ, USA, AF-100-15). For differentiation, ReN VM mitoKeima cells were cultured in media lacking EGF and FGF, but containing 1 mM dibutyryl-cAMP (Invivochem, Libertyville, IL, USA, V1846) and 2 ng/mL glial-derived neurotrophic factor (GDNF, Peprotech, 450-10). After two weeks of differentiation, ReN VM mitoKeima cells were treated with 20 µM carbonyl 3-chlorophenylhydrazone (CCCP, Sigma, C2759) or DMSO vehicle (Sigma, D4540). The media used to treat the differentiated neurons contained B-27 without antioxidants (Thermo Fisher Scientific, 10889038). Fibroblasts were treated with DMSO or valinomycin (Cayman Chemical, Ann Arbor, MI, USA, 10009152). All cells were maintained at 37 °C and 5% CO_2_ in a humidified atmosphere.

### 2.3. Genome-Editing with CRISPR-Cas9

Hetero- and homozygous p.R275W ReN mitoKeima cells were generated by CRISPR-Cas9. The guideRNA targeting exon 7 of the *PRKN* gene (GTGTGACAAGACTCAATGAT) was used in combination with a single-stranded oligodeoxynucleotide containing the desired base change as template for homologous directed repair. A silent blocking mutation was used to prevent re-editing by Cas9. For the generation of heterozygous cells, we mixed the mutant with a wild-type repair template that only contained the blocking mutation. Sanger sequencing of single clones was used to confirm correct gene-editing. The absence of unwanted mutations was tested by sequencing the loci of the five most likely off-target sites as nominated by Benchling Biology software (Version 2021, retrieved from www.Benchling.com, accessed on 24 September 2021).

ReN mitoKeima PRKN KO cells were generated using lentiviral delivery of a plasmid encoding Cas9 and a sgRNA targeting the *PRKN* gene close to the translational start site. The target sequence (AGTGACCATGATAGGTACGT) was cloned into the BsmI site of pLenti CRISPR V2. LentiCRISPR v2 was a gift from Feng Zhang (Addgene, Watertown, MA, USA, 52961). Lentiviral particles were produced using standard protocols and incubated with ReN mitoKeima cells in a serial dilution. Transduced cells were selected with 0.2 µg/mL puromycin (Invitrogen, A1138-03) and had a 90% reduction of PRKN protein levels. Single clones were isolated and the absence of PRKN was confirmed by Western blot.

### 2.4. Western Blot

Cells were washed two times with cold PBS and harvested in RIPA buffer (50 mM Tris [Sigma-Aldrich, 648311], pH 8.0, 150 mM NaCl [Sigma Aldrich, S5886], 0.1% SDS [Fisher Scientific, BP166-500], and 0.5% Deoxycholate [Sigma-Aldrich, D6750], 1% NP-40 [Sigma-Aldrich, I3021]), supplemented with a protease (Sigma-Aldrich, 11697498001) and phosphatase inhibitor (Sigma-Aldrich, 04906837001) cocktail. Samples were spun (21,000× *g*, 4 °C, 15 min), the supernatant saved, and protein concentration was measured (Thermo Fisher Scientific, 23225). Cell lysates and human brain SDS fraction were diluted in Laemmli buffer (62.5 mM Tris, pH 6.8, 1.5% SDS, 8.33% glycerol [Fisher Scientific, BP2291], 1.5% β-mercaptoethanol [Sigma, M3148], and 0.005% bromophenol blue [Sigma, B5525]). SDS-PAGE was performed with 8-16% Tris-Glycine gels (Invitrogen, XP08165BOX) using standard conditions. Proteins were blotted onto polyvinylidene fluoride membranes (Thermo Fisher Scientific, IPVH00010). Membranes were washed in TBST (50 mM Tris, pH 7.4, 150 mM NaCl, 0.1% Tween-20 [Sigma, P1379]), blocked in 5% dry milk powder (Sysco, Houston, TX, USA, 5398953) in TBST, and incubated with primary antibodies overnight. Vinculin or GAPDH antibodies were only incubated for 30 min at RT. Membranes were washed three times with TBST before secondary antibodies were incubated for 1 h at room temperature. Signal was visualized using Immobilon Western Chemiluminescent HRP Substrate (Millipore Sigma, WBKLS0500) and recorded with a Chemidoc MP imaging system (Bio-Rad, Hercules, CA, USA).

The following antibodies were used for Western blot: anti-PRKN 5C3 (mouse IgG1; BioLegend, San Diego, CA, USA, 865602), anti-PRKN PRK8 (mouse IgG2b; Millipore, MAB5512, used for the solubility analysis), anti-PINK1 (mouse IgG1; BioLegend, DU46-1.1), anti-pS65-Ub (rabbit; CST, Danvers, MA, USA, 62802), anti-MFN2 (mouse IgG2a; Abcam, Cambridge, UK, ab56889), anti-VCL (mouse; Sigma, V9131), and anti-GAPDH (mouse; Meridian, Aberdeen, WA, USA, H86504M). Where indicated, subtype-specific mouse secondary antibodies were used to enhance the signal. The following secondary antibodies from Jackson ImmunoResearch (West Grove, PA, USA) were used: donkey anti-mouse IgG [715-035-150], donkey anti-rabbit IgG [711-035-152], goat anti-mouse IgG1 [115-035-205], goat anti-mouse IgG2a [115-035-206], and goat anti-mouse IgG2b [115-035-207].

### 2.5. RNA Analysis

Total RNA was extracted using a RNeasy spin mini kit (Qiagen, Germantown, MD, USA, 74104). A one-step quantitative reverse transcription PCR (BioRad, Hercules, CA, USA, 1725151) was set up using 50 ng of total RNA on a 384-well LightCycler 480 instrument (Roche Diagnostics, Rotkreuz, Switzerland). Relative expression levels of *PINK1* and *PRKN* were determined using RPL27 as housekeeping gene [35] Values were normalized to the relative expression level of the WT control. Primer sequences used were: Human *PRKN* (5′-GCTGTGGGTTTGCCTTCT-3′, 5′-TCCACTGGTACATGGCAGC-3′), human *RPL27* (5′-GATCGCCAAGAGATCAAAGATAAAA-3′, 5′-CTGAAGACATCCTTATTGACGACAGT-3′).

### 2.6. Immunofluorescence

Cells were cultured and differentiated as stated previously. Cells were treated, then fixed by adding 4% paraformaldehyde (Sigma, 441244) for 10 min, washed with PBS, and permeabilized with 1% Triton X-100 (Sigma, X100) in PBS for 10 min. Cells were blocked with 10% goat serum (Invitrogen, 16210072) in PBS for 1 h. Then, primary antibodies were added in a solution of 1% BSA in PBS for 60 min. After washing two times and blocking again with 10% normal goat serum in PBS for five min, secondary antibodies were added in a solution of 1% BSA in PBS for 60 min. Next, cells were washed, and Hoechst reagent (Invitrogen, H21492) was added in PBS for five min. Finally, cells were washed and fixed again with 4% in PBS. Antibodies used and their respective concentration: pS65-Ub (CST, 62802, 1:1000) coupled with goat anti-rabbit Alexa488 (Molecular probes, A11034, 1:1000), LAMP2 (DSHB, Iowa City, IA, USA, H4B4-c, 1:1000) coupled with goat anti-mouse Alexa568 (Molecular probes, Eugene, OR, USA, A11004, 1:1000), HSP60 (Arigo, Zhubei, Taiwan, ARG10757, 1:2000) coupled with goat anti-chicken Alexa647 (Molecular probes, A21449, 1:1000). Cells were imaged using AxioObserver (Zeiss, Oberkochen, Germany) with Zen Blue software (version 6.1.7601 Service Pack 1 Build 7601).

### 2.7. Sandwich ELISA

Meso Scale Discovery (MSD) ELISA against pS65-Ub was performed as described previously with modifications [36]. Briefly, 96-well plates (Meso Scale Diagnostics, L15XA-3) were coated with pS65-Ub antibody (CST, 62802) in 200mM sodium carbonate buffer pH 9.7 overnight. Wells were blocked the next day (1% BSA in TBST) and lysates were added for 1 h at room temperature. After three wash steps with wash buffer (TBST), total Ub antibody (ThermoFisher, 14–6078–82) was added for 2 h at room temperature, followed by sulfo-tag labeled anti-mouse secondary antibody (Meso Scale Diagnostics, R32AC-1). Signal was measured in MSD Gold Read buffer (Meso Scale Diagnostics, R92TG-2) using a MESO QuickPlex SQ 120 instrument (Meso Scale Diagnostics, Rockville, MD, USA). Final antibody concentrations were 1 µg/mL for all steps.

### 2.8. Flow Cytometry

Cells were detached in Accutase (Innovative Cell Technologies, San Diego, CA, USA, S1067) and centrifuged (220× *g*, 5 min). The pellet was washed twice in DPBS (Invitrogen, 14190250) before resuspending in 1 mL flow buffer (1× HBSS [Invitrogen, 14065–056], 10 mM HEPES [Sigma, H4034–100G], 2% BSA). A volume of 1 µL SYTOX Red dead cell stain (Thermo Fisher Scientific, S34859) was added to each sample to permit live/dead discrimination. Samples were kept on ice before analysis by flow cytometry. Analysis of mitoKeima was performed using an Attune NxT flow cytometer (Thermo Fisher Scientific, Waltham, MA, USA). The 405 nm excitation laser with 603/48 nm emission (standard VL3 detector optical path) was used to detect neutral Keima. Acidic mitoKeima signal was detected using the 561 nm excitation and a custom optical path for the YL2 detector as described previously [37]. Height signals were analyzed in log scale. Data were analyzed in FCS Express (Version 6, De Novo Software). Events were gated for a main population, standard doublet discrimination, and dead cell exclusion via SYTOX Red. Acidic/neutral mitoKeima ratios were determined for each live event and the geometric mean of the ratio was used to statistically analyze several experiments.

### 2.9. Protein Sequential Extraction

As a readout of PRKN solubility, cells were harvested in 1% Triton-X-100 (Sigma, X100-500ML) in PBS with protease and phosphatase inhibitor cocktails. Samples were centrifuged (136,000× *g*, 30 min, 4 °C) and the supernatant was collected as the Triton-X-soluble fraction. The pellet was washed in 1% Triton-X-100 buffer two times, discarding the supernatant each time. Then, the pellet was resuspended in the same volume of 2% SDS in PBS, centrifuged (136,000× *g*, 30 min, 4 °C), and the Triton-insoluble/SDS-soluble fraction was collected. The protein concentration of both fractions was measured via BCA Protein assay, and concentration values from the Triton-X-soluble fraction were used for sample preparation for both soluble and insoluble fractions before Western blot.

### 2.10. Human Brain Samples

Frozen midfrontal cortex samples (180–200 mg) were homogenized with a Dounce tissue grinder (DWK, Millville, NJ, USA, K885300-0002) in 5 volumes of Tris-buffered saline (TBS; 50 mM Tris, 150 mM NaCl, pH 7.4) containing phosphatase and protease inhibitors cocktails. 5× RIPA buffer was added and incubated at 4 °C for 30 min with rotation. Samples were then spun at 100,000× *g* for 60 min at 4 °C. The supernatant (referred to as ‘RIPA soluble fraction’ or ‘soluble’) was collected. The residual pellet was washed with 1x RIPA buffer twice and spun again at 100,000× *g*. The pellet was resuspended in 2% SDS with phosphatase and protease inhibitors, sonicated ten cycles (30 s ON, 30 s OFF with high power level) in a Bioruptor sonication system (Diagenode, Liege, Belgium), then boiled at 95 °C for 5 min. The samples were then spun at 100,000× *g* for 60 min at 22 °C and the supernatant was used as ‘2% SDS fraction’ or ‘insoluble fraction’.

### 2.11. Statistical Analysis

Data analysis and visualizations were performed using GraphPad Prism (version 10.2). Quantifications are expressed as mean ± SEM for cell data or as median ± interquartile range for brain data. Statistical comparisons for cell data were performed via one- or two-way ANOVA with Tukey’s post hoc test, as indicated in the figure legends. Comparison of brain samples was performed with a non-parametric Mann–Whitney test (* *p* < 0.05, ** *p* < 0.01, *** *p* < 0.001).

## 3. Results

### 3.1. Structural Analysis of PRKN p.R275W

**Figure 1:**
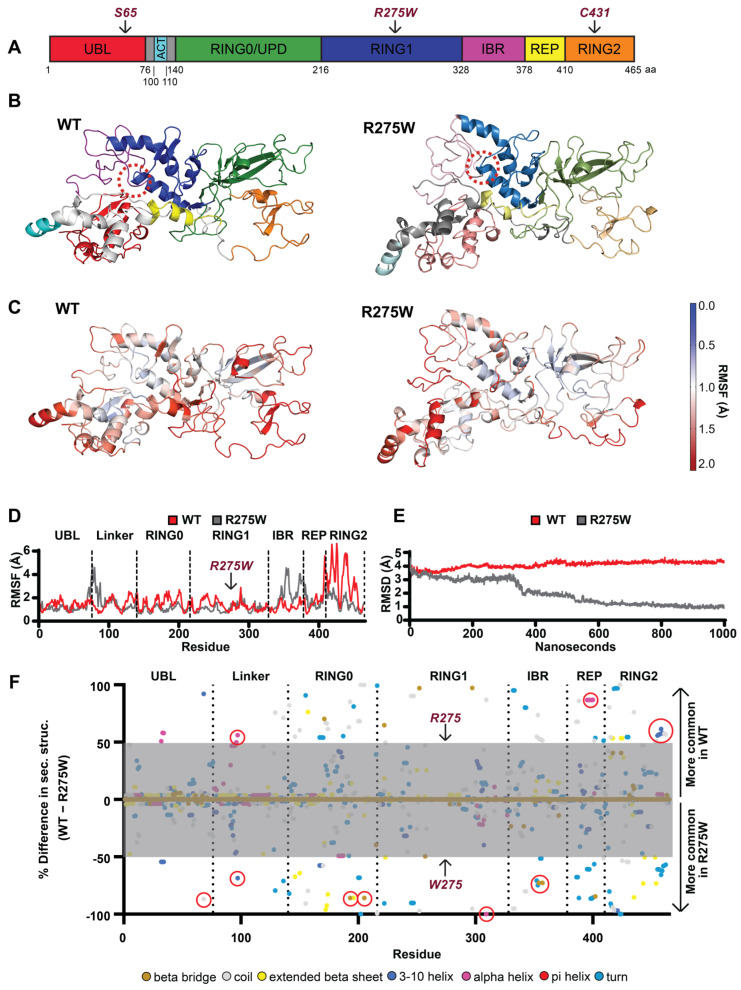
Structure of PRKN WT and p.R275W and conformational changes. (**A**) Schematic overview of the PRKN protein structure and individual domains: Ub-like domain (UBL, red), linker domain (gray), activating element (ACT, cyan), really interesting new gene domain (RING0, green), RING1 (blue), in-between-RING domain (IBR, purple), repressor element of PRKN (REP, yellow), and RING2 (orange). (**B**) A 3D model of the autoinhibited structure of PRKN WT and p.R275W with individual domains colored as in (**A**); the location of position 275 is highlighted by a red dashed circle. (**C**) A 3D heat map illustration of the root mean square fluctuation (RMSF) for PRKN WT and p.R275W over the course of the simulations. (**D**) Comparison of RMSF for each residue of PRKN WT (red) and PRKN p.R275W (gray). Domains are labeled and separated with dotted lines. (**E**) Root mean square deviation (RMSD) of PRKN WT (red) and PRKN p.R275W (gray) structure over 1 µs simulation. (**F**) Per-residue difference plot of the secondary structure averaged over the entire simulation and colored by secondary structure feature. Shown are percent occupancy times of each residue in a specific secondary structure for PRKN WT (top) and PRKN p.R275W (bottom). Differences greater than 50% (outside the gray area) are considered most meaningful. Secondary structure colors are indicated at the bottom of the figure.

We recently generated a model of the closed conformation of PRKN WT [26] that was built on the available crystal structure(s) of PRKN and contains all domains of the E3 ubiquitin ligase (Figure 1A). For this study, we introduced the p.R275W mutation into this model and performed simultaneous 1 µs length unbiased all-atom molecular dynamics simulations (MDS) of PRKN WT and p.R275W. The simulations allow the structures to explore a variety of conformations over the duration of the simulation (Figure S1A,B). The most common conformations exhibited by PRKN WT (Figure 1B) and PRKN p.R275W (Figure 1C) show visible difference (for superposition see Figure S1C). A zoom into the respective local interactions around residue 275 is shown in Figure S1D,E. First, we calculated the per-residue root mean square fluctuations (RMSF) in atomic positions, which describes the magnitude of motion each residue experiences throughout the course of the simulation. For an overview, we heat-mapped the RMSF onto the 3D model of PRKN WT and p.R275W. Our analysis shows that the majority of the p.R275W undergoes lower magnitude motions throughout the simulation, as displayed by the intensification of the red scale in the WT model, versus the relative tempering into the white and blue scale in the p.R275W model (Figure 1C). The per-residue RMSF is 2D mapped with domains indicated for reference. This reveals that the motion suppression is present in all three RING domains (RING0, -1, and -2), while the linker, IBR, and part of the REP possess elevated motion amplitudes (Figure 1D). Examining the global dynamics by averaging across the entire molecule using root mean square deviation in atomic positions (RMSD) confirms an overall suppression of motions in the PRKN p.R275W structure (Figure 1E). However, we found large conformational differences between the structures, with an RMSD of 5.38Å between the most common conformations Figure 1C and Figure S1C). This is indicative of structural disturbances caused by the variant.

To investigate those structural differences further, we analyzed the secondary structure for each residue across the simulation. To compare PRKN WT and PRKN p.R275W, we calculated the percent difference in occupancy time for each secondary structure form for each residue (Figure 1F). There was a shift from well-defined turn to disordered coil in the tail of the UBL in PRKN p.R275W (Figure 1F, indicated by a red circle near the dashed line between UBL-linker). While the linker is partially disordered, portions of it developed alpha-helices (H dots), which appear to have led to a relative suppression of motion locally in the WT; however, in PRKN p.R275W, this helicity was rarer, as seen in the increase of shorter helices (G dots), giving rise to greater magnitude motions in the linker (Figure 1F, red circled structures in the linker region). We believe these could signal a UBL-linker junctional destabilization. In the RING0 domain of p.R275W, short beta bridges formed between residues C196 and S205 (Figure 1F, circles in RING0, near residue 200). The RING1 domain of PRKN p.R275W showed a perturbation of the helix 3 region from L301 to E309, fluctuating between alpha and 3–10 helical character (Figure 1F, circle near residue 300). Again, in the IBR domain of p.R275W, short beta bridges formed between C352 and G357 (Figure 1F, red circle in the IBR domain). These beta bridges possibly contributed to the rigidification of the RING domains and transmission of motion through helical and sheet features within these domains. Also, the REP domain of p.R275W had a marked loss in alpha helical character from E395 to A401 (Figure 1F, circle in the REP region); this likely contributed to destabilization of the REP domain. The RING2 domain of PRKN WT showed a prevalent 3–10 helix from p.R455 terminating at p.H461 that was lost in PRKN p.R275W (Figure 1F, circle in RING2). The combined effects seen in the PRKN p.R275W structure are consistent with a disruption of the closed PRKN conformation. From our simulations we calculated the total potential energy of PRKN WT and R275W, which were −4513.83 kcal/mol and −4420.21 kcal/mol, respectively. The difference in stability on the 1 μs timescale suggests that R275W is considerably more susceptible to non-enzymatic degradation, i.e., unfolding. From the perspectives both of causing conformational differences and of modifying the potential energy of protein, the p.R275W appears to destabilize PRKN.

### 3.2. Skin Fibroblasts with PRKN p.R275W Substitution Have Reduced PRKN Protein Levels

To analyze PRKN p.R275W functionally on the endogenous level, we first compared skin fibroblasts from p.R275W mutation carriers to controls. We were able to obtain healthy cultures of skin fibroblasts from two affected individuals with heterozygous p.R275W mutation and three from early-onset PD patients with compound heterozygous p.R275W mutations. Fibroblasts from age- and sex-matched unaffected individuals without PRKN mutations were used as control cells (Table 1).

**Figure 2:**
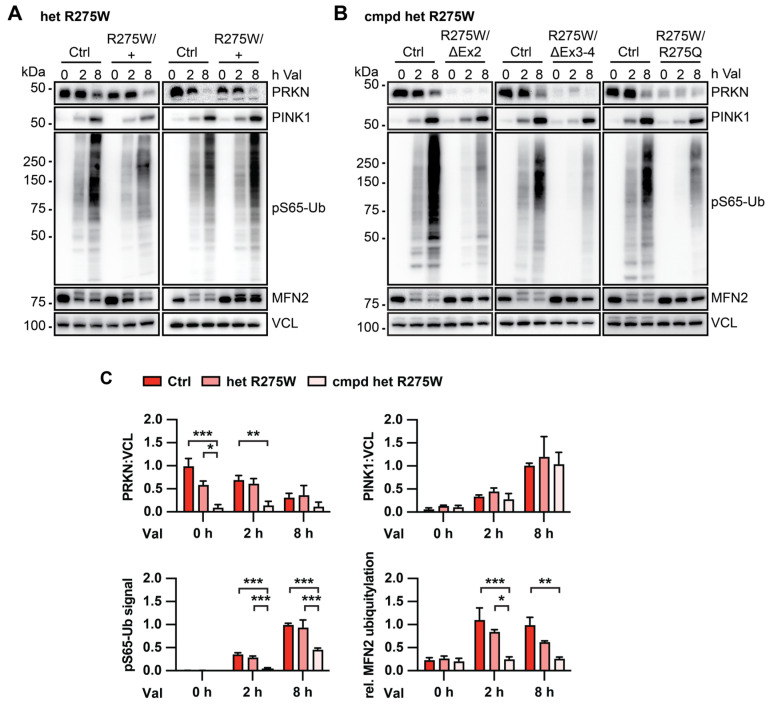
Biochemical analysis of PINK1-PRKN signaling in human fibroblasts with or without the PRKN p.R275W mutation. Dermal fibroblasts from controls and PD patients with heterozygous (**A**) or compound heterozygous (**B**) PRKN p.R275W mutation were treated with 1 μM valinomycin (Val) for 0, 2, or 8 h. Cell lysates were collected for Western blot analysis and probed with antibodies against PRKN, PINK1, pS65-Ub, and MFN2. VCL was used as loading control. (**C**) Densitometric analysis of PRKN and PINK1 Western blot signals normalized to VCL. Relative modification of MFN2 was calculated as the ratio of the upper (ubiquitylated) to lower (unmodified) MFN2 band. Quantification of pS65-Ub levels measured by sandwich ELISA. Data were normalized to control cells, either at the 0 or the 8 h time point. Data shown are mean ± SEM (n = 4 for WT, n = 2 for p.R275W heterozygous, and n = 3 for compound heterozygous). Statistical analysis was performed by two-way ANOVA followed by Tukey’s post hoc test (* *p* < 0.05, ** *p* < 0.01, *** *p* < 0.001). Only statistically significant comparisons are indicated.

To induce the PINK1-PRKN signaling pathway, cells were treated with the mitochondrial depolarizer valinomycin, then subjected to Western blot analysis. In WT control cells, valinomycin treatment induced the stabilization of PINK1 protein, and the formation of pS65-Ub, the joint product of PINK1-PRKN. This was accompanied by an increase in the ubiquitylation of MFN2, a representative substrate of PRKN [38]. The signal of the PRKN protein itself decreased upon valinomycin treatment, similar to what we and others have observed in response to several mitophagy inducers. Compared to controls, cells from heterozygous PRKN p.R275W carriers showed somewhat reduced PRKN protein levels in untreated cells and PRKN levels further decreased over time of treatment. Compared to treated control cells, at least one fibroblast line with heterozygous PRKN also showed slightly reduced pS65-Ub levels. Both lines showed a trend towards less MFN2 ubiquitylation, while PINK1 levels remained similar (Figure 2A).

Cells from individuals with compound heterozygous p.R275W mutations showed a larger difference to control cells. All three lines had strongly reduced PRKN protein levels, and robustly reduced pS65-Ub levels compared to control cells. Consistent with a complete loss of PRKN activity, we also observed the absence of MFN2 ubiquitylation in these cells (Figure 2B). We next confirmed the observed effects by quantification of the Western blots. To measure pS65-Ub, a sensitive mesoscale discovery (MSD) sandwich enzyme-linked immunosorbent assay (ELISA) was used [36]. Quantification of the data confirmed that total PRKN protein levels, pS65-Ub, and MFN2 ubiquitylation were all significantly lower in cells from compound heterozygous p.R275W carrier. Although not statistically significant, PRKN and modified MFN2 showed a trend for lower levels even in cells with heterozygous p.R275W PRKN substitution, while pS65-Ub levels were undistinguishable from controls (Figure 2C).

### 3.3. PRKN p.R275W Presents with Substantially Reduced Protein Levels in Isogenic DA Neurons

**Figure 3:**
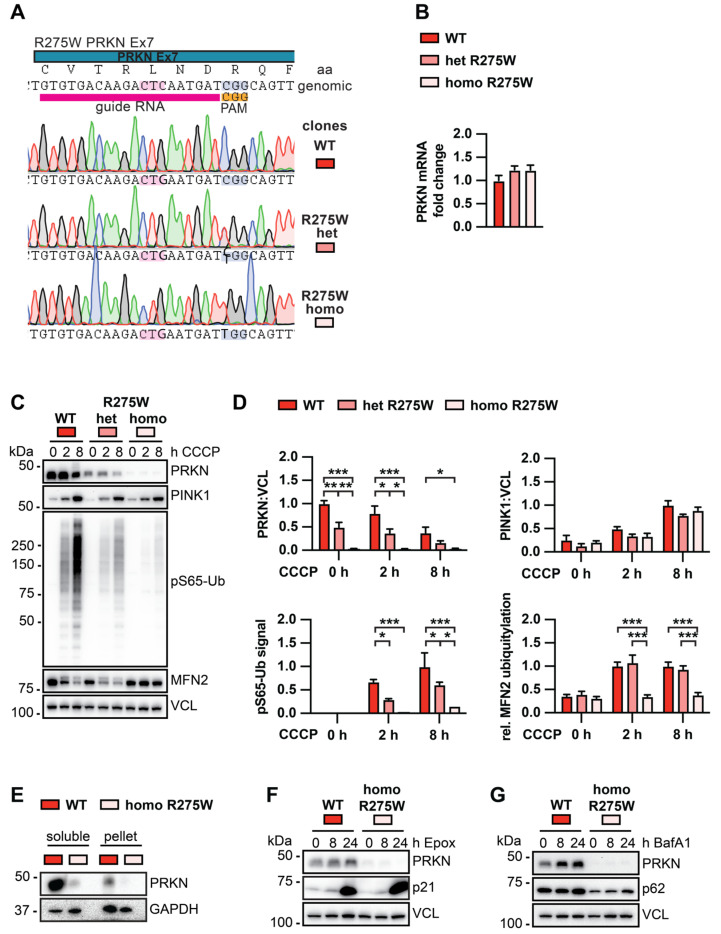
Biochemical analysis of PINK1-PRKN signaling in isogenic DA neurons with or without the PRKN p.R275W mutation. (**A**) Schematic of CRISPR–Cas9 target location, gRNA, and results from Sanger sequencing of isogenic clones. (**B**) PRKN mRNA levels in WT, p.R275W heterozygous, and p.R275W homozygous gene-edited DA neurons. Data shown are mean ± SEM (n = 3). (**C**) WT, p.R275W heterozygous, and p.R275W homozygous gene-edited DA neurons were treated with 20 µM CCCP for 0, 2, or 8 h. Cell lysates were collected for Western blot analysis and probed with antibodies against PRKN, PINK1, pS65-Ub, MFN2, and VCL. Representative Western blot shows levels of PRKN, PINK1, pS65-Ub, and MFN2 for all three cell lines and treatments. (**D**) Densitometric analysis of PRKN and PINK1 normalized by VCL. Quantification of pS65-Ub protein levels by sandwich ELISA. Relative modification of MFN2 was calculated as the ratio of the upper (ubiquitylated) to lower (unmodified) MFN2 band. Statistical analysis was performed via two-way ANOVA (* *p* < 0.05, ** *p* < 0.01, *** *p* < 0.001) and data shown are mean ± SEM (n = 3). Only statistically significant comparisons are indicated. (**E**) Proteins from WT and PRKN p.R275W homozygous gene-edited DA neurons were sequentially extracted in two fractions for solubility analysis and probed with antibodies against PRKN and GAPDH. (**F**) Gene-edited DA WT neurons and PRKN p.R275W homozygous DA neurons were treated with 200 μM epoxomicin for 0, 8, or 24 h to test PRKN degradation via the proteasome. Cell lysates were collected for Western blot analysis and probed with antibodies against PRKN, p21, and VCL. (**G**) Gene-edited DA neurons were treated with 400 μM bafilomycin A1 for 0, 8, or 24 h to test for PRKN levels upon inhibition of the autophagosome-lysosome fusion. Cell lysates were collected and probed against PRKN, p62, and VCL.

To investigate PRKN p.R275W in cells with the same genomic background, we generated isogenic clones with a hetero- or homozygous PRKN p.R275W substitution by CRISPR–Cas9 (Figure 3A). As a parental cell line, we used midbrain-derived neuronal precursors [39] that had been transduced with lentivirus to express mitochondrially targeted Keima red protein (mitoKeima), a mitophagy reporter [37]. Briefly, the same guideRNA targeting exon 7 of *PRKN* was used in both cases but was delivered with different ssDNA repair templates for insertion of the p.R275W substitution into one or both alleles. Cells were transfected and single clones were carefully screened for on- and off-target effects by Sanger sequencing. Per genotype, we confirmed three independent clones, and used them as biological replicates for functional analysis upon differentiation into dopaminergic (DA) neurons [37,39].

Quantitative RT-PCR showed that *PRKN* mRNA levels were not significantly altered between WT and hetero- or homozygous p.R275W DA neurons (Figure 3B). However, compared to WT neurons, protein levels of PRKN were genotype-dependently reduced in cells with hetero- or homozygous PRKN p.R275W mutation (Figure 3C,D). Treatment with the mitochondrial uncoupler carbonyl cyanide *m*-chlorophenyl hydrazone (CCCP) further revealed a dose-dependent reduced induction of pS65-Ub. The extent of the pS65-Ub levels seen in the homozygous p.R275W neurons was similar to a complete knock-out (KO) of PRKN, suggesting that the remaining pS65-Ub is due to PINK1 activity only (Figure S2). There was no difference regarding the stabilization of PINK1 between neurons with different genotypes, suggesting that the reduced induction of pS65-Ub was mostly PRKN-dependent. A defect in the ubiquitylation of MFN2 could only be observed in homozygous but not heterozygous PRKN p.R275W neurons.

Several groups including ours have reported that PRKN p.R275W when overexpressed in cells forms aggregates, suggesting this mutation renders the protein insoluble [19,20,22,40]. To test the possibility that endogenous PRKN p.R275W shifts into the insoluble fraction, we re-extracted the cell pellets with 2% SDS. However, while we indeed found a small proportion of PRKN WT in the pellet fraction, we were not able to detect PRKN p.R275W in the same fraction (Figure 3E). To determine whether PRKN p.R275W was degraded via the ubiquitin proteasome or the autophagy pathway, we pharmacologically inhibited either pathway in the differentiated neurons. Treatment with the proteasome inhibitor epoxomicin led to a drastic increase of the proteasomal substrate p21; however, levels of PRKN p.R275W remained unchanged during the treatment (Figure 3F). Treatment with the autophagy–lysosome fusion inhibitor bafilomycin A1 also failed to increase levels of PRKN p.R275W (Figure 3G), suggesting that neither pathway alone is responsible for the degradation of the mutant PRKN protein. It is possible that PRKN is degraded redundantly by multiple pathways and that they must be inhibited simultaneously to observe a stabilization of PRKN.

### 3.4. PRKN p.R275W Fails to Mediate Mitophagy in Dopamine Neurons

**Figure 4:**
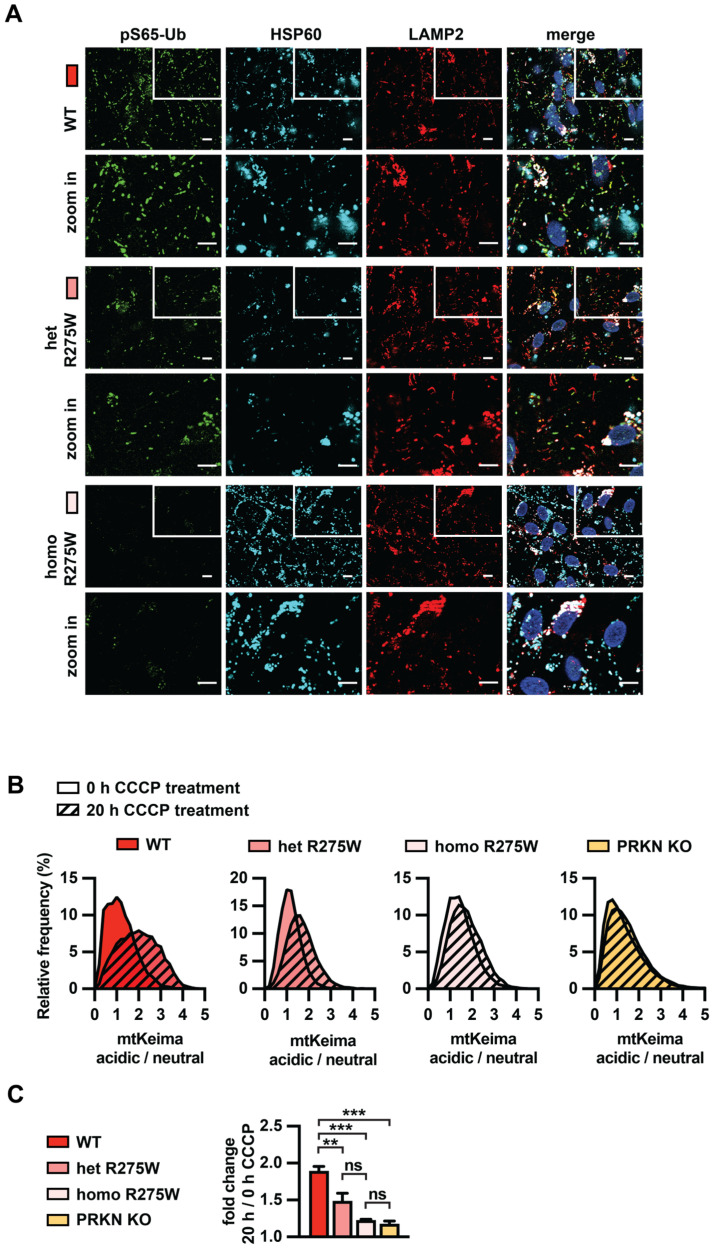
Analysis of PINK1-PRKN mitophagy execution in p.R275W isogenic cell lines. (**A**) Immunofluorescence imaging for gene-edited DA neurons. WT, p.R275W hetero- or homozygous cells were treated with 20 µM CCCP for 8 h. Cells were stained with antibodies against pS65-Ub (green), lysosomal marker LAMP2 (red), and mitochondrial marker HSP60 (cyan). Hoechst was used to stain nuclei. Scale bar = 10 μm. (**B**) Quantification of mitophagy in WT, PRKN p.R275W heterozygous or homozygous neurons, and PRKN KO using flow cytometry of mitoKeima. Representative frequency distribution of acidic/neutral mitoKeima in untreated cells and the shift that is observed after 20 h CCCP treatment (shaded) are shown. (**C**) Fold change of the acidic/neutral mitoKeima ratio at 20 h CCCP treatment compared to untreated cells for each cell line. Data shown are mean ± SEM (n = 3). Statistical analysis was performed via one-way ANOVA (** *p* < 0.01, *** *p* < 0.001, ns = non-significant).

We next studied the initiation and execution of mitophagy in PRKN p.R275W DA neurons. Immunofluorescence staining showed that upon treatment with CCCP, pS65-Ub accumulated in WT cells and to a weaker extent in heterozygous p.R275W cells, but was strongly reduced in homozygous p.R275W cells (Figure 4A), consistent with the results from the Western blot and MSD. We further analyzed the pH-sensitive ratiometric fluorescent mitoKeima probe that the neurons stably expressed in flow cytometry experiments. The excitation spectrum of Keima shifts to longer wavelengths as the protein enters an acidic environment [41]. This can be used as a readout for mitophagy [41,42]. Consistent with our previous report [37], WT neurons showed a clear shift towards more acidified mitochondria upon CCCP treatment (Figure 4B). Neurons heterozygous for p.R275W showed a reduced shift, and in homozygous p.R275W neurons the acidification was even further reduced, similar to PRKN KO neurons (Figure 4B,C). Together, our data suggest that p.R275W is a loss-of-function allele and contributes to early-onset PD risk via impairments of protein stability and mitophagy.

### 3.5. PRKN Protein Levels Are Reduced in Human Autopsy Brain from p.R275W Mutation Carriers

**Figure 5:**
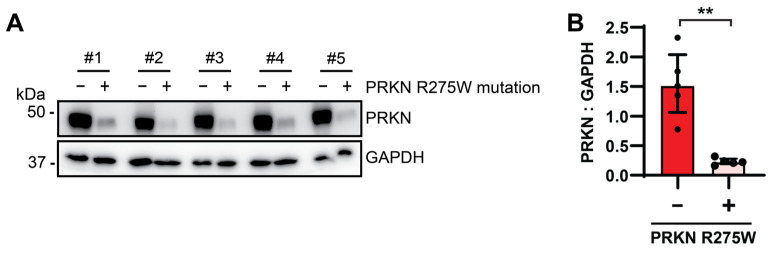
PRKN p.R275W levels are decreased in human post-mortem brain samples. (**A**) Age-, sex- and disease-matched human post-mortem brain samples were lysed for Western blot analysis and probed with antibodies against PRKN and GAPDH. (**B**) Densitometric analysis of PRKN normalized to GAPDH. Data shown are median ± interquartile range (IQR) (n = 5). Statistical analysis was performed using the Mann–Whitney test (** *p* < 0.01).

To ascertain our findings from cultured patients’ cells and gene-edited dopaminergic neurons with the PRKN p.R275W mutant in culture, we next turned to human post-mortem brain tissue. Given that homozygous or compound heterozygous PRKN mutations are extremely rare, we selected cases with a heterozygous p.R275W mutation and paired these with age-, sex-, and disease-matched samples (Table 2) with similar extent of alpha-synuclein (LBD type), tau (Braak stage), and amyloid-beta (Thal phase) pathologies. Midfrontal cortex tissue was obtained from all cases with or without the PRKN p.R275W mutation, and proteins were sequentially extracted. PRKN protein was only detectable in the SDS-soluble fraction and consistent with findings in vitro, and the levels of PRKN were strongly and significantly reduced in autopsy brain from p.R275W substitution carriers. The soluble fraction was used to probe GAPDH as a loading control (Figure 5). Altogether, our analyses of fibroblasts, dopamine neurons, and brain tissue demonstrate reduced PRKN protein levels of the p.R275W loss-of-function mutation.

## 4. Discussion

Mutations in the *PRKN* gene are the most common genetic cause of early-onset PD and are thought to impair the mitophagy process, a critical mitochondrial quality control pathway [11]. When mitochondria are damaged, the E3 Ub ligase PRKN is structurally de-repressed, enzymatically activated, and recruited from the cytosol by the Ub kinase PINK1. Both enzymes then jointly decorate damaged mitochondria with pS65-Ub to facilitate their selective elimination by the autophagy–lysosome system. Herein we investigated the structural and functional consequences of the most frequent missense variant in *PRKN* (c.823C  >  T, rs34424986). The pathogenic p.R275W mutation is located in the RING1 domain of PRKN close to the E2 co-enzyme binding site. Point mutations in PRKN can typically be categorized into different dysfunctional classes and p.R275W has been described as activation impaired and defective in mitophagy [2,3,25,46]. However, several unusual phenotypes have also been reported for the p.R275W variant as it has been suggested to retain residual enzymatic activity in vitro, to cause aggresome formation in cells, or to directly exert neurotoxicity in vivo in flies [19,20,47]. While numerous studies have overexpressed the p.R275W mutation, to the best of our knowledge, this is the first to focus on endogenously expressed PRKN p.R275W in two independent cell models and in human post-mortem brain samples. In addition to these functional analyses, we also performed molecular dynamics simulations to study the protein structure of PRKN p.R275W over time.

The PRKN protein transitions from a closed, auto-inhibited structure to an activated, pS65-Ub bound state upon which the Ub-like domain is released and phosphorylated by PINK1 at serine-65. This leads to larger structural rearrangements in PRKN that enable binding of an Ub-loaded E2 co-enzyme and enable the accessibility of the catalytic center C431 (reviewed in [24]). The structural analysis of PRKN p.R275W in the activated state has already indicated a disrupted interaction with the helix that mediates pS65-Ub binding [25]. Here, we performed long (1 µsec) molecular dynamics simulation of the p.R275W mutation compared to PRKN WT, starting from the closed, inactive structure of PRKN. In order to achieve comparable results that are not based on random fluctuations, we performed these simulations side-by-side. Structurally, we observed signatures of destabilization of the PRKN closed conformation from our energetics measurements, as well as elevated dynamics and secondary structure differences in the repressive regions, UBL and linker domains. Collectively, our computational structural analyses suggest that p.R275W might affect the stability of the PRKN protein, consistent with a very recent study that systematically investigated a large number of *PRKN* mutations [25].

We next analyzed early-onset PD patients’ fibroblasts with a PRKN p.R275W substitution compared to cells from unaffected age- and sex-matched controls. The cells were treated with valinomycin to damage mitochondria and activate the PINK1-PRKN pathway and pS65-Ub was measured as the joint PINK1-PRKN product. We also monitored MFN2 ubiquitylation as a direct substrate of PRKN. Patients’ cells from carriers with compound heterozygous p.R275W substitution showed a clear mitophagy deficit and displayed strongly reduced pS65-Ub induction and MFN2 ubiquitylation. All three compound heterozygous fibroblast lines had much reduced PRKN protein level compared to controls, which was most apparent in untreated cells, suggesting that the absence of PRKN at steady-state might contribute to the loss-of-function phenotype upon valinomycin treatment. It is noteworthy that the p.R275W/p.R275Q line showed slightly higher PRKN protein levels than the other two compound heterozygotes that contain exon deletions, suggesting the p.R275Q mutation might be slightly more stable than p.R275W, consistent with findings from a recent deep mutational scanning of *PRKN* [48].

In heterozygous p.R275W cells, the same effects seemed present, but they were much less pronounced and not statistically significant with the two heterozygous lines that we had access to. It is possible that the effects would become significant with a larger sample size. Alternatively, the absence of an effect of heterozygous p.R275W in fibroblasts could be reflective of the low penetrance of heterozygous p.R275W mutations [49,50]. In fact, it is still very much a matter of debate whether heterozygous PRKN mutations cause or increase the risk of PD [51,52,53]. While the analysis on the population levels does not seem to support a clear role of heterozygous *PRKN* mutations, on the individual level it has been shown that the loss of one *PRKN* allele causes dopamine deficits in the brain consistent with mild, preclinical symptoms [54,55,56,57,58]. However, *PRKN* is an unusually large gene, and a potential second cryptic mutation may exist on the other allele and must be excluded for each case. Yet, it is also possible that a monoallelic *PRKN* mutation could predispose an individual but alone is not sufficient to cause disease [59]. Whether or not an individual with a heterozygous *PRKN* mutation might develop clinical PD might very much depend on the levels of mutant versus WT *PRKN* expression, concomitant protein levels, and enzymatic activity of the vital E3 Ub ligase.

Similar to fibroblasts, DA neurons also showed a 50% reduction of PRKN protein levels in heterozygous p.R275W cells. However, in these isogenic cells, we observed clear functional deficits with regards to pS65-Ub levels and neuronal mitophagy rates as determined by mitoKeima. This could be due to higher levels of PRKN in DA neurons compared to fibroblasts [60]. Indeed, we have previously shown that the increase of PRKN during differentiation leads to a strong amplification of the pS65-Ub levels [37]. Alternatively, the larger effect size of PRKN reduction in DA neurons might be explained by other cell-type specific differences. The isogenic background of the DA neurons further helps to keep the variation low and allows to easier discovery of genotype effects. Nevertheless, while the detection of functional phenotypes in heterozygotes may depend on the cell type as well as nature and timing of the analyses along an enzymatic cascade, the disease risk of an individual may critically depend on expression levels of functional PINK1 and PRKN, as well as the extent and duration of stress and damage that activates the pathway, more than genetic status alone. Additional work is warranted on heterozygotes, but importantly, our data classify p.R275W as a complete loss-of-function mutation. Despite some residual PRKN protein, p.R275W is functionally indistinguishable from a full PRKN KO in DA neurons with regard to defects in pS65-Ub production, MFN2 ubiquitylation, and mitophagy execution upon mitochondrial uncoupling.

Previous studies that analyzed overexpressed PRKN p.R275W have repeatedly shown the appearance of inclusions in transfected cells [19,20,21,22]. A shift to the insoluble fraction has also been reported. However, we could not detect endogenous p.R275W in the insoluble fraction of cultured DA neurons, suggesting PRKN p.R275W does not become insoluble when expressed endogenously in cells. Nevertheless, an increased propensity of PRKN p.R275W to form aggregates compared to PRKN WT is still possible and may play a role in the brain, where PRKN tends to become insoluble with age, especially in the substantia nigra possibly as the result of reactive dopamine metabolites and increased oxidative stress [61]. However, in frontal cortex, PRKN protein levels were strongly reduced in the SDS-soluble fraction from p.R275W carrier consistent with the general instability of the mutant. Nevertheless, it is interesting to note that PRKN p.R275W, in contrast to other mutations, seems to be more commonly associated with LBs (reviewed in [62]). However, this notion could also be skewed by the higher frequency of the p.R275W variant compared to other mutations and by the overall low number of cases that were autopsied so far. It will be important to determine if this holds true as more PRKN cases are being autopsied. However, it has earlier been speculated that PRKN activity may be needed for LB formation, and more recently also that these structures may sequester material undegradable by autophagy [63]. In line with this idea, LBs were found to contain mainly lipid membranes and crowded organelles including mitochondrial remnants and were also closely associated with pS65-Ub [64,65]

Despite the instability of the p.R275W mutant protein and its consequent loss of function, therapeutics opportunities may exist, nevertheless. A recent report showed that genetic or pharmacological inhibition of the deubiquitylating enzyme USP30 can effectively modulate mitophagy and restore the haploinsufficiency in p.R275W heterozygous fibroblasts, as measured by an increase in pS65-Ub levels and in MFN2 ubiquitylation [66,67]. Notably though, the rescue effect was due to modulation of events upstream and mediated in part through the remaining WT *PRKN* allele instead of rescuing the pathogenic phenotype of the p.R275W mutant protein itself. Another report showed that introduction of “activating” mutations that were designed to structurally impede the auto-inhibitory interactions within PRKN can also increase mitophagy execution in cells overexpressing p.R275W [25]. Here, the rescue effect was obtained by modulating the activation deficits of the pathogenic variant, yet without affecting mutant protein levels. In the future, stabilization of p.R275W protein and prevention of its degradation with chemical chaperone combined with an activating compound may not only restore PRKN protein levels but may also further boost the remaining enzymatic activity of the mutation to therapeutically relevant levels, thus providing a novel avenue of intervention.

## 5. Conclusions

Here we investigated the structure and function of the most frequent PRKN missense mutation p.R275W. Molecular dynamics simulations of the p.R275W substitution revealed global structural changes indicative of reduced stability of mutant PRKN protein. In cell models with endogenous PRKN p.R275W expression and in human post-mortem brain samples, we further found strongly reduced mutant PRKN protein levels. Cells with PRKN p.R275W showed less substrate modification and lower mitochondrial turnover when challenged with stress. Collectively, our data suggest that under endogenous conditions the p.R275W mutation causes a loss of function by destabilizing the PRKN protein.

## Figures and Tables

**Table 1:** List of fibroblasts from controls and PD patients with PRKN p.R275W substitution.

Cell Line	*PRKN* Genotype	Age at Onset	Age at Biopsy	Sex
Ctrl 1	WT	n/a	55	F
Ctrl 2	WT	n/a	67	F
Ctrl 3	WT	n/a	64	M
Ctrl 4	WT	n/a	46	M
Het 1	R275W/+	43	61	F
Het 2	R275W/+	44	48	M
Cmpd Het 1	R275W/ΔEx2	25	51	M
Cmpd Het 2	R275W/ΔEx3-4	37	72	M
Cmpd Het 3	R275W/R275Q	47	51	F

Ctrl: control; Het: heterozygous; Cmpd Het: compound heterozygous; n/a: not applicable; F/M: female/male.

**Table 2:** List of human post-mortem brain samples without/with the PRKN p.R275W substitution.

Pair	p.R275W Substitution	Age	Sex	Pathological Diagnosis	Braak Stage	Thal Phase
#1	−	71	M	iLBD	III	2
	+	72	M	DLBD	II	0
#2	−	85	M	AD, DLBD	IV	5
	+	94	M	DLBD	IV	2
#3	−	86	F	AD	V	3
	+	88	F	AD	V	n/a
#4	−	90	F	AD	VI	5
	+	97	F	AD	VI	5
#5	−	93	F	AD	V	5
	+	99	F	AD	V	n/a

Sex: F/M—female/male; pathological diagnosis, iLBD: incidental Lewy body disease, DLBD: diffuse Lewy body disease [43], AD: Alzheimer’s disease; Braak stage: Braak neurofibrillary tangle stage [44]; Thal phase: Thal amyloid stage [45], n/a: not available.

## Data Availability

The original contributions presented in the study are included in the article and Supplementary Material; further inquiries can be directed to the corresponding author(s).

## Supplementary Materials

The following supporting information can be downloaded at: https://www.mdpi.com/article/10.3390/cells13181540/s1, Figure S1: Global and local conformational comparisons of PRKN WT and p.R275W; Figure S2: Comparison of PRKN p.R275W with PRKN KO.

## Abbreviations

CCCP	carbonyl cyanide *m*-chlorophenyl hydrazone
DA	dopamine
ELISA	enzyme-linked immunosorbent assay
KO	knock-out
LB	Lewy body
MFN2	mitofusin 2
MSD	Meso Scale Discovery
MitoKeima	mitochondrially targeted Keima red protein
PD	Parkinson disease
pS65-Ub	phosphorylated ubiquitin at Serine 65
RING	really interesting new gene
Ub	ubiquitin
WT	wild-type
