# Supplementary material for: Structural and Functional Characterization of the Most Frequent Pathogenic PRKN Substitution p.R275W
Source: Cells. 2024 Sep 13;13(18):1540. doi: 10.3390/cells13181540 (PMC11430725; doi:10.3390/cells13181540)

Structural and functional characterization of the most frequent pathogenic *PRKN* substitution p.R275W

Bernardo A Bustillos[1,#], Liam T Cocker[1,#], Mathew A Coban[1], Caleb A Weber[1], Jenny M Bredenberg[1], Paige K Boneski[1], Joanna Siuda[2], Jaroslaw Slawek[3,4], Andreas Puschmann[5], Derek P Narendra[6], Neill R Graff-Radford[7], Zbigniew K Wszolek[7], Dennis W Dickson[1,8], Owen A Ross[1,8], Thomas R Caulfield[1,9,10,11,12], Wolfdieter Springer[1,8]*, and Fabienne C Fiesel[1,8]*

[1] Department of Neuroscience, Mayo Clinic, Jacksonville, FL 32224, USA
[2] Department of Neurology, Faculty of Medical Sciences in Katowice, Medical University of Silesia, 40-055 Katowice, Poland
[3] Department of Neurology, St. Adalbert Hospital, 80-462 Gdansk, Poland
[4] Division of Neurological and Psychiatric Nursing, Faculty of Health Sciences, Medical University of Gdansk, 80-210 Gdansk, Poland
[5] Department of Neurology, Lund University, Skane University Hospital, Sweden
[6] Inherited Movement Disorders Unit, Neurogenetics Branch, National Institute of Neurological Disorders and Stroke (NINDS), NIH, Bethesda, Maryland, USA.
[7] Department of Neurology, Mayo Clinic, Jacksonville, FL 32224, USA
[8] Neuroscience PhD Program, Mayo Clinic Graduate School of Biomedical Sciences, Jacksonville, FL, 32224, USA
[9] Department of Neurosurgery, Mayo Clinic, Jacksonville, FL 32224, USA.
[10] Department of Cancer Biology, Mayo Clinic, Jacksonville, FL 32224, USA.
[11] Department of Biochemistry & Molecular Biology, Mayo Clinic, Jacksonville, FL 32224, USA.
[12] Department of Computational Biology, Mayo Clinic, Jacksonville, FL 32224, USA.
[#] These authors contributed equally to this work
* Correspondence: springer.wolfdieter@mayo.edu (W.S.); fiesel.fabienne@mayo.edu (F.C.F.)

Supplemental Figure S1. Global and local conformational comparisons of PRKN WT and p.R275W.

(**A**) Superposition of the 7 conformations PRKN WT adopts throughout the course of the simulation, clustered via the QT clustering algorithm. (**B**) Superposition of the 5 conformations PRKN p.R275W adopts throughout the course of the simulation. (**C**) Superposition of the most common conformation of PRKN WT and p.R275W from their respective simulations. (**D**) Zoom into PRKN WT and the respective local interactions within 5 Å. (**E**) Zoom into p.R275W and the respective local interactions within 5 Å.

Supplemental Figure S2. Comparison of PRKN p.R275W with PRKN KO

(**A**) WT, p.R275W heterozygous, p.R275W homozygous and PRKN KO gene-edited DA neurons were treated with 20 μM CCCP for 0, 2, or 8 h. Cell lysates were collected for western blot analysis and probed with antibodies against PRKN, PINK1, pS65-Ub, MFN2 and VCL.

Supplemental Figure S1

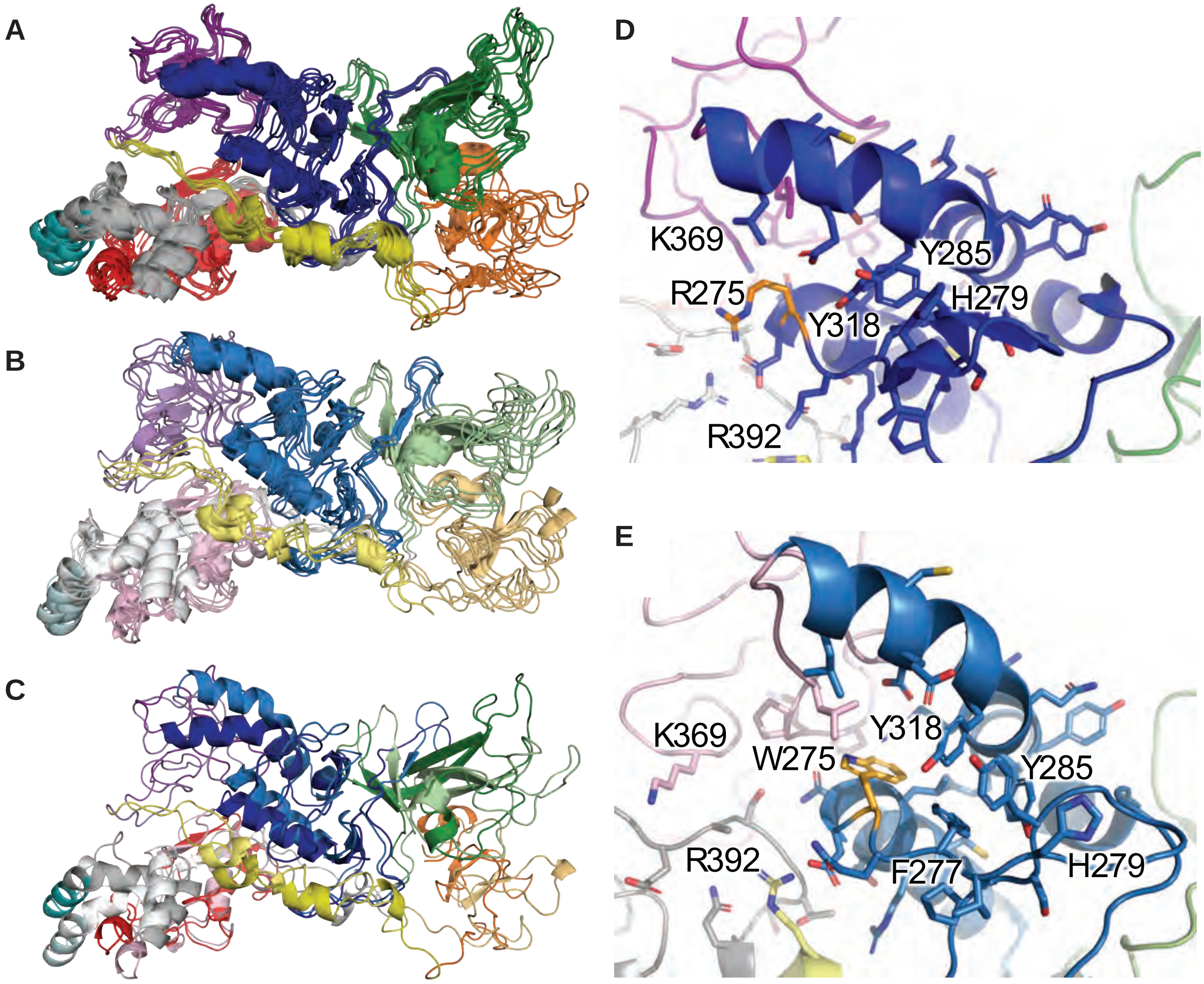

Supplemental Figure 2

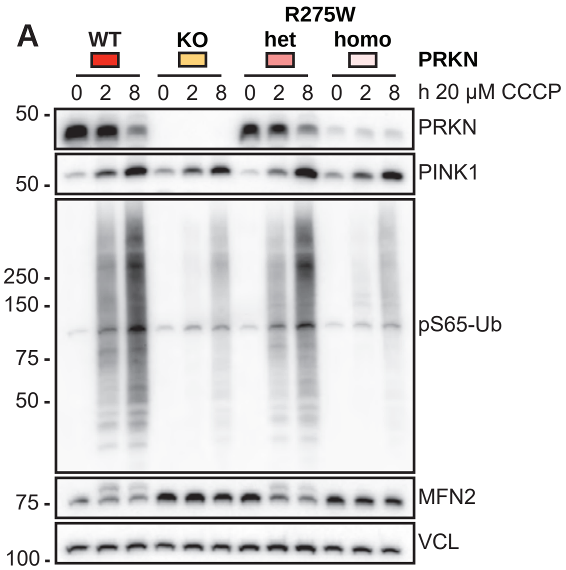

Western blot images to Bustillos, Cocker *et al.*

Figure 2

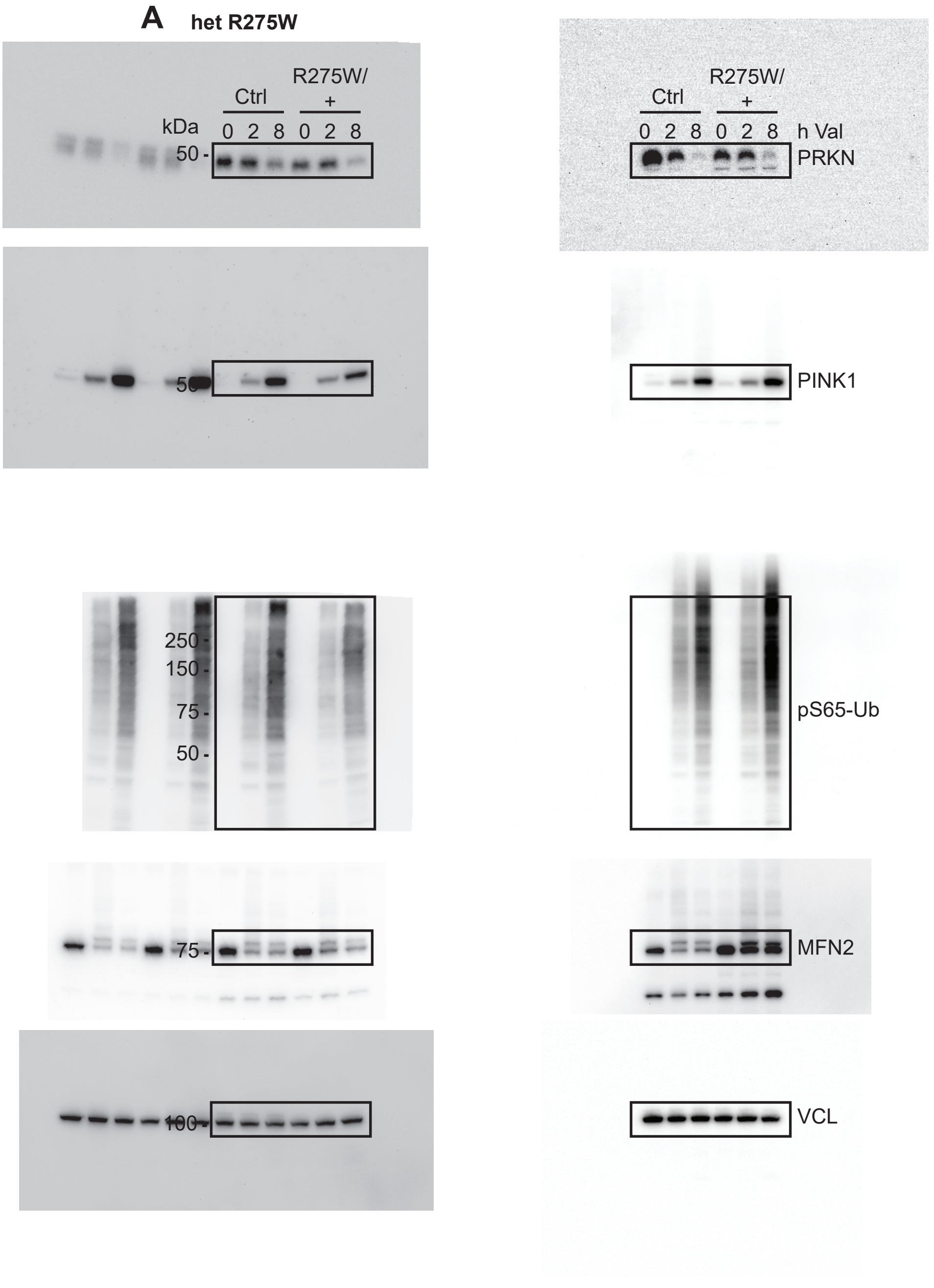

Western blot images to Bustillos, Cocker *et al.*

Figure 3

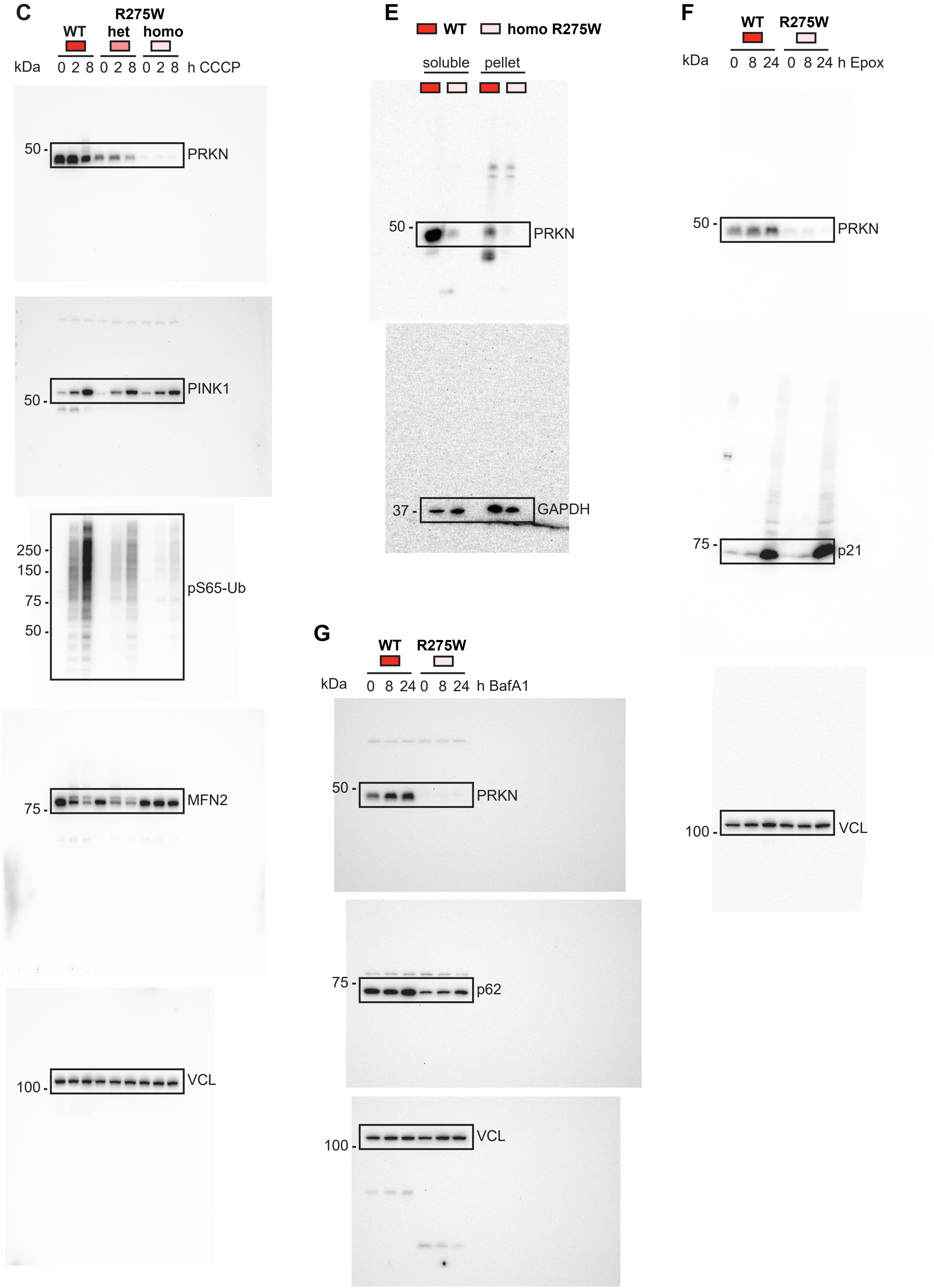

Western blot images to Bustillos, Cocker *et al.*

Figure 5

A

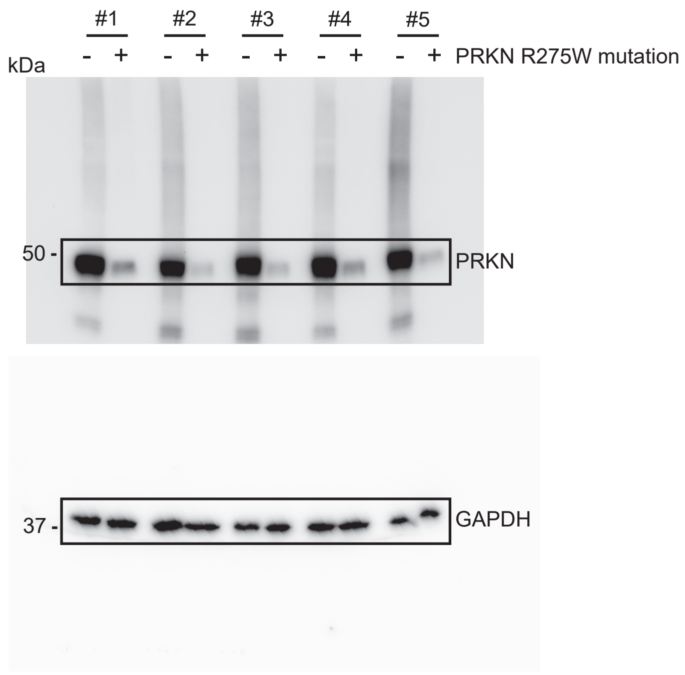

Western blot images to Bustillos, Cocker *et al.*

Supplemental Figure S2

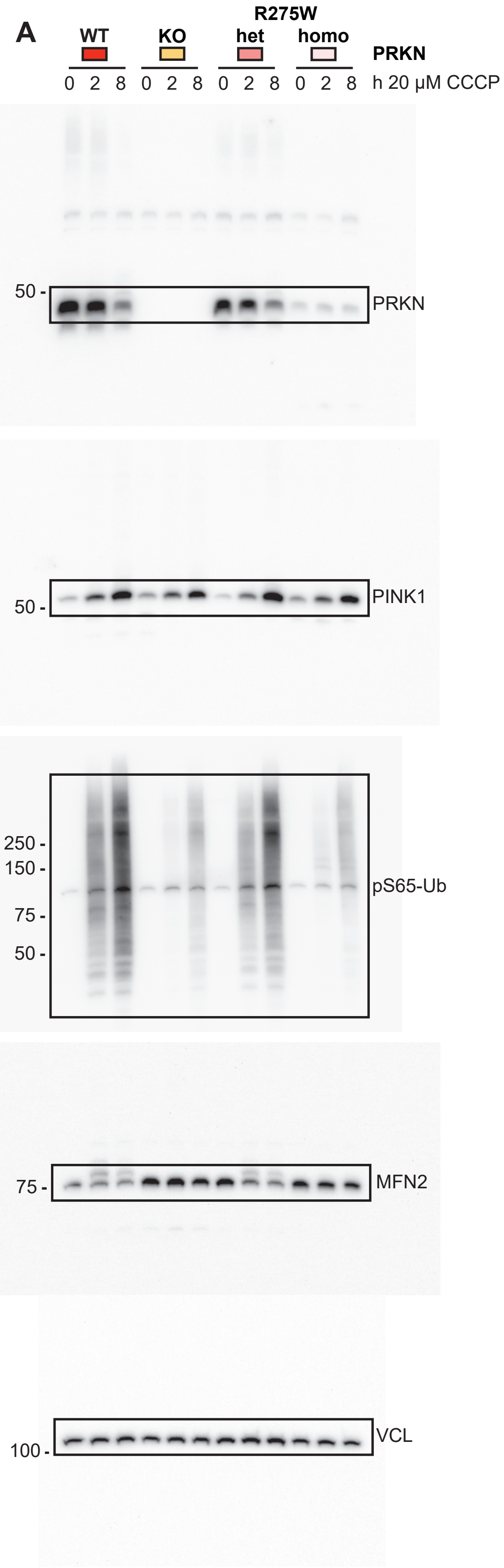

Western blot images to Bustillos, Cocker *et al.*

Figure S2 continued

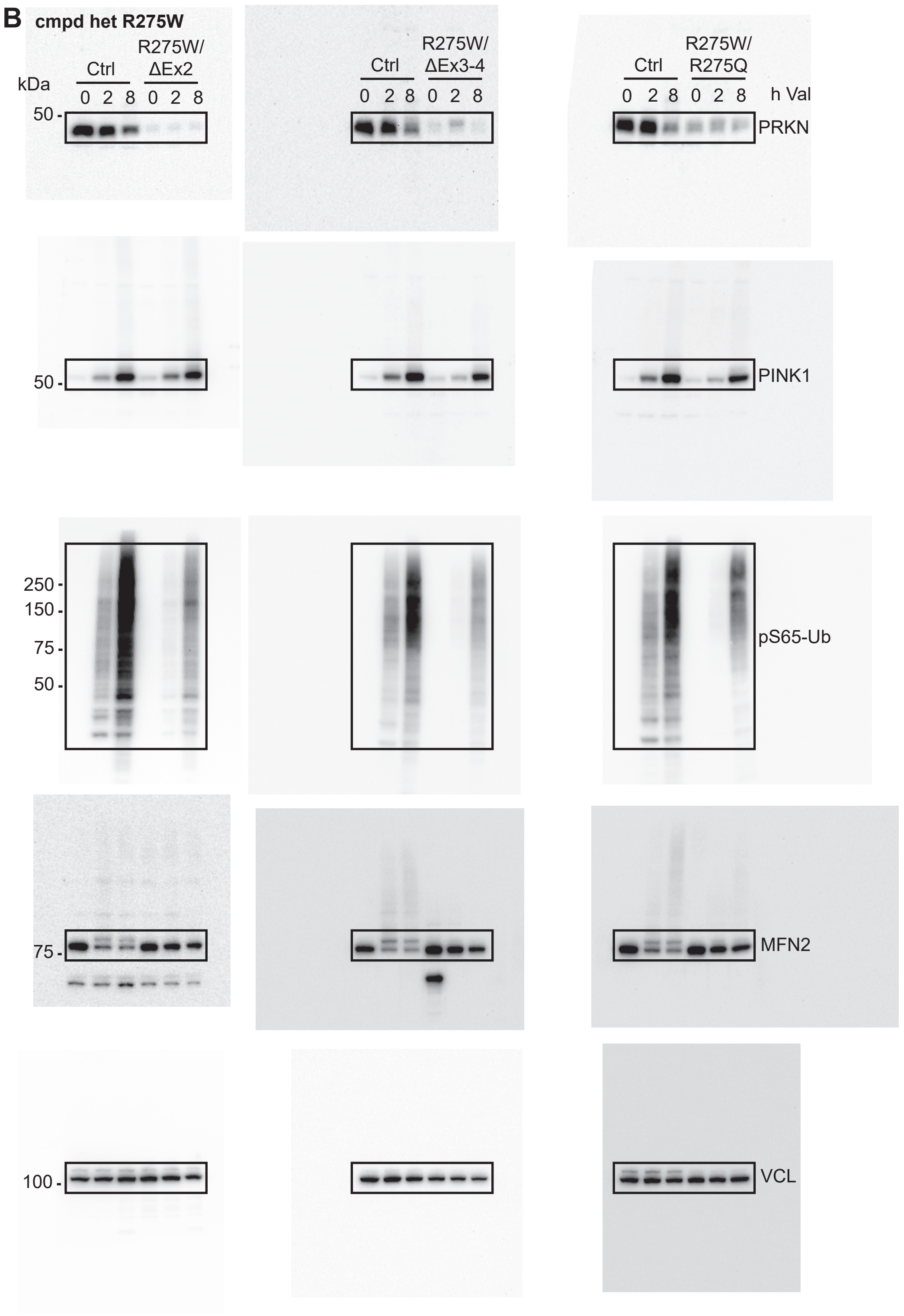

Supplement: Supplementary file 1 [file cells-13-01540-s001.zip › cells-3133081-supplementary.pdf]
